# The Effects of Applied Potential and Carbon Donor on Succinic Acid Production via Electro-Fermentation

**DOI:** 10.3390/microorganisms14030686

**Published:** 2026-03-18

**Authors:** Jingjing Wang, Wenqiao Yuan

**Affiliations:** Department of Biological and Agricultural Engineering, North Carolina State University, Raleigh, NC 27695, USA

**Keywords:** applied potential, electro-fermentation, succinic acid, *Actinobacillus succinogenes*

## Abstract

This study was conducted to understand how applied potential modulates metabolic flux toward succinic acid during xylose electro-fermentation by *Actinobacillus succinogenes* under varying feed concentrations (15, 20, 25 g/L). Electro-fermentations were conducted with applied potential at −1.5 V and −2.5 V and compared to open circuit control. Product distribution and carbon balance were quantified to assess the effect of potential on pathway routing. Results showed that applied potential consistently reduced formic acid and increased succinic acid selectivity. At 20 g/L xylose, the highest succinic acid yield was 0.80 mol/mol at −2.5 V, a 28.88% increase compared to that of the control (0.62 mol/mol). Formic acid and acetic acid yields were 0.73 and 0.60 mol/mol, representing a 48.83% and 16.09% reduction, respectively. The carbon allocation to succinic acid was 51% with a total carbon recovery of 81%. In addition, the effects of 10 g/L and 15 g/L NaHCO_3_, as well as 10 g/L NaHCO_3_ supplemented with gaseous CO_2,_ were evaluated at 15 g/L xylose and −2.5 V. Supplementation with gaseous CO_2_ increased succinic acid yield from 0.74 to 0.85 mol/mol and improved total carbon recovery from 75% to 84%. Collectively, these findings show that applied potential, in combination with bicarbonate or CO_2_ supply, can be strategically employed to improve succinic acid production.

## 1. Introduction

Succinic acid (SA) is a four-carbon dicarboxylic acid recognized as one of the U.S. Department of Energy’s “Top 12 Platform Chemicals” derived from biomass [1]. Its industrial applications are diverse, including biodegradable polymers and resins, pharmaceutical formulations, food additives, and a variety of other value-added products [2]. Conventional petrochemical synthesis routes are considered unsustainable due to their reliance on fossil carbon and high energy demands. Therefore, biological succinic acid fermentation by natural producers has been extensively studied, with *Actinobacillus succinogenes*, *Mannheimia succiniciproducens*, *Basfia succiniciproducens*, and *Anaerobiospirillum succiniciproducens* being the most used strains. At bench scale, reported succinate yields were approximately 0.49 to 0.92 g/g, depending strongly on carbon source, CO_2_ availability, pH control, and reactor operation mode (batch, fed-batch, or continuous) [3]. Among these microorganisms, *A. succinogenes* is regarded as a particularly promising succinate producer due to its high production capacity and ability to utilize a broad spectrum of carbon sources, including C5 and C6 sugars, glycerol, and biomass-derived substrates. It is commonly cultivated under anaerobic or microaerobic conditions at near-neutral pH with CO_2_ supplementation to promote carboxylation reactions and enhance succinate formation, features that make it well-suited for industrial applications [4]. Despite these advantages, succinate production by *A. succinogenes* remains constrained by intracellular redox imbalance, competition for carbon flux among succinate and byproduct pathways, and limited substrate utilization efficiency under practical operating conditions [5]. To overcome these challenges, various biotechnological strategies, such as metabolic engineering, process optimization, and systems biology, have been investigated to enhance yield and process efficiency [6,7,8,9].

More recently, microbial electrochemical technologies (METs) have emerged as a promising alternative, offering a means to steer microbial metabolism through electrochemical control [10]. METs encompass microbial fuel cells (MFCs), microbial electrolysis cells (MECs), microbial electrosynthesis (MES), and electro-fermentation (EF), each designed for distinct applications [11,12,13]. Among these, EF represents a promising strategy to improve conventional fermentation by modifying reaction rates, product yields, titers, and distribution [14]. Under EF, electrodes are introduced as additional redox partners: they either act as an electron donor, acceptor, or are used to regulate the oxidation-reduction potential of the system [15,16]. By coupling microbial metabolism to an electrode, EF enables external control over intracellular electron fluxes, cofactor regeneration, and redox balance. In SA fermentation, particularly, applying potentials at the cathode can drive metabolic pathways toward more reduced products, thereby enhancing SA formation [17]. The earliest evidence of *A. succinogenes* utilizing electrically supplied reducing power to support growth and drive metabolism dates back to 1999, demonstrating the fundamental feasibility of electrochemical assistance [18]. More recent studies reported that EF increased succinate yield by approximately 18%, from 0.64 to 0.76 g/g, and increased succinate titer from 12.05 to 16.48 g/L under electrochemically reduced conditions [19]. Similarly, another study showed that modulation of electrode potential increased succinate yield to 0.747 g/g, representing a 15.65% improvement compared with fermentation without electrochemical assistance [20]. Beyond pure sugar substrates, EF has also been applied to lignocellulosic feedstocks; using corncob hydrolysate, succinate titers increased 1.31-fold and intracellular reducing power increased 1.33-fold relative to non-electro controls, achieving 18.1 g/L succinate with a yield of 0.60 g/g sugar under −1.8 V, demonstrating the feasibility of integrating EF with biomass-derived substrates [21]. However, the extent to which applied potential interacts with substrate concentration to regulate SA production by *A. succinogenes* remains underexplored. This is particularly relevant for lignocellulosic bioprocessing, where xylose is an abundant but underutilized sugar, and process optimization requires understanding how substrate behaves under electrochemical control.

To address this gap, the present study evaluated how applied potential influences substrate concentration on SA production in a two-electrode EF system. The use of a two-electrode configuration, comprising a cathode as the working electrode and an anode as the counter electrode, was chosen for its simplicity and suitability for process-level investigations. This approach allows systematic evaluation of SA yield and titer under different electrochemical and substrate conditions, providing practical insight into process performance. The results from this study thus establish how substrate concentration and applied potential jointly shape SA production.

## 2. Materials and Methods

### 2.1. Microorganism and Cultivation

*Actinobacillus succinogenes* 130Z (ATCC 55618) was obtained from the American Type Culture Collection (ATCC, Manassas, VA, USA) and revived following the supplier’s instructions. The strain was maintained on Trypticase Soy Agar composed of (per liter): tryptone 17 g, soytone 3 g, dextrose 2.5 g, NaCl 5 g, K_2_HPO_4_ 2.5 g, and agar 15 g. For inoculum preparation, a single colony was transferred into Trypticase Soy Broth, prepared by omitting agar from the TSA formulation, and incubated overnight at 37 °C with shaking at 200 rpm.

For batch cultivation, the fermentation medium contained 100 mM phosphate buffer (pH 7.0), 5 g/L yeast extract, and a defined concentration of xylose depending on the experimental design. To provide inorganic carbon, 10 or 15 g/L (~120 or 180 mM) NaHCO_3_ was included, with or without additional CO_2_ supplementation. NaHCO_3_ served both as an inorganic carbon source for microbial metabolism and as a buffering agent for pH control. The selected NaHCO_3_ concentrations were based on a previous work that demonstrated increased succinate production at NaHCO_3_ concentrations ≥ 75 mM [22]. All chemicals used were purchased from Fisher Scientific (Waltham, MA, USA).

For the CO_2_ supplementation experiments, pure CO_2_ was supplied at 0.1 vvm during the first 12 h of fermentation. Prior to entering the reactor, the gas passed through a bottle containing sterilized water to humidify the gas and minimize evaporation from the culture. The reactor headspace was connected to a sterile 0.2 μm filter to allow gas release while maintaining sterile conditions and preventing pressure buildup.

### 2.2. Electro-Fermentation System Setup and Operation

**Figure 1:**
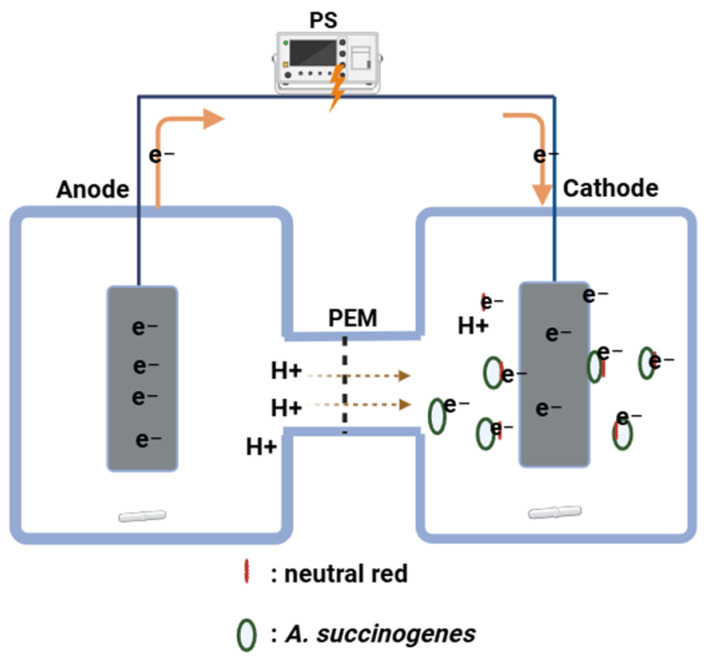
Schematic of the electro-fermentation system integrating an anode and a cathode chamber separated by a proton exchange membrane (PEM). Electrons generated at the anode are transferred through the external circuit under power supply (PS) control and delivered to the cathode. Neutral red (red bars) acts as a redox mediator, shuttling electrons from the cathode to *A. succinogenes* cells (green circles), where they drive reductive metabolism. Protons (H^+^) generated in the anode migrate across the PEM to maintain charge balance.

A dual-chamber EF system was constructed using two glass bottles connected by 40 mm diameter glass flanges (Adams & Chittenden Scientific Glass, Berkeley, CA, USA), secured with chemical-resistant seals and wraparound knuckle clamps. Each chamber had a total volume of 330 ± 5 mL, consisting of a liquid working volume of 270 mL (including the electrode) and a headspace of 60 mL. The two chambers were separated by a proton exchange membrane (PEM; PFSA D 170-U, Fuel Cell Store, Fuel Cell Earth LLC, College Station, TX, USA), which permitted selective proton transfer while preventing bulk mixing of electrolytes. Graphite felt was used as the electrodes in both chambers. The construction is shown in Figure 1.

The PEM was pretreated by sequential boiling in 30% (*v*/*v*) hydrogen peroxide and deionized water, followed by soaking in 0.5 M sulfuric acid and deionized water for one hour each to ensure activation and removal of contaminants. The membrane material was selected for its chemical resistance and long-term stability under acidic and electrochemical conditions.

Graphite felt electrodes (G475, AvCarb Material Solutions, Lowell, MA, USA) were pretreated by immersion in 0.1 N hydrochloric acid for 24 h to remove surface impurities, then rinsed and stored in deionized water until use. Each graphite felt electrode (3 cm × 4 cm, 0.8 cm thickness) was placed in its respective electrolytic chamber and connected to an external DC power supply (1501s, Yihua Electronic Equipment, Guangzhou, China). A copper wire was affixed to the electrode and connected to the power supply cable via an alligator clip, with the wire externally insulated to prevent contact with the electrolyte solution.

The catholyte consisted of the fermentation medium described previously for batch cultivation, while the anolyte was 100 mM phosphate buffer (pH 7.0). To enhance ionic conductivity, the anolyte was supplemented with 0.1 M NaCl. Neutral red was added to the catholyte as a redox mediator at a final concentration of 0.1 mM after sterile filtration through a 0.22 μm membrane filter.

All experiments were conducted in the H-shaped dual-chamber reactors (Figure 1) maintained at 37 ± 2 °C using a temperature-controlled water bath system (Julabo, 9116000, Allentown, PA, USA). Both chambers were stirred continuously at 200 rpm using magnetic stirrers (Cimareci Poly 15, Thermo Fisher Scientific, Waltham, MA, USA) to ensure homogenous mixing and mass transfer. The potential was maintained at fixed levels of 1.5 V or 2.5 V, depending on the experimental condition. These voltages were applied using a DC power supply by connecting the positive and negative terminals to the anode and cathode, respectively. In this configuration, the cathode was 1.5 V or 2.5 V more negative than the anode. Therefore, the applied voltage was represented as −1.5 V or −2.5 V relative to the anode. As a control, a parallel system with the same electrode configuration was operated under open-circuit conditions, with no external voltage applied.

### 2.3. Metabolites Analysis

Cell growth was monitored by measuring optical density (OD) at 600 nm using a microplate reader (BioTek Instruments, Inc., Winooski, VT, USA). The pH was monitored at each sampling point using a calibrated pH meter (Denver UB 10, Denver Instrument, Bohemia, NY, USA).

The concentrations of xylose, SA, formic acid (FA), and acetic acid (AA) were determined using high-performance liquid chromatography (HPLC, Shimadzu Corporation, Kyoto, Japan), equipped with both UV/VIS detector (SPD-20A, Shimadzu, Japan) and refractive index detectors (RID-10A, Shimadzu Corporation, Kyoto, Japan). The system was operated at a detection temperature of 40 °C. Separation was carried out using an Aminex HPX-87H column (300 × 7.8 mm, Bio-Rad, Hercules, CA, USA), maintained at 50 °C. The mobile phase was 0.005 M H_2_SO_4_, delivered at a flow rate of 0.5 mL/min. Lactic acid, pyruvic acid, and propionic acid were not measured because they are not considered major extracellular end products of xylose fermentation by *A. succinogenes* under succinate-producing conditions. In this organism, carbon flux is primarily directed toward succinic acid, with acetic acid and formic acid being the dominant byproducts. Lactate is typically detected only at trace levels under CO_2_^−^ or bicarbonate-buffered conditions, while pyruvate is a central metabolic intermediate that does not usually accumulate extracellularly, and propionate formation is negligible due to the lack of a dominant propionate-producing pathway [6,22]. For these reasons, they were not prioritized for quantification in this study.

Carbon distribution and total carbon recovery were calculated using Equations (1) and (2). The amount of inorganic carbon was determined from the known initial concentration of NaHCO_3_ added to the medium, assuming one mole of inorganic carbon per mole of NaHCO_3_. End-point dissolved inorganic carbon was not measured; therefore, in the carbon recovery and carbon distribution calculations, C_(NaHCO3)_ represents the initial inorganic carbon supplied, not the residual inorganic carbon remaining at the end of fermentation. Carbon recovery is thus reported relative to the total carbon input (consumed xylose plus added inorganic carbon).

(1) $$Total\;carbon\;recovery\left(\%\right)=100\times \frac{4{∆C}_{SA}+2{∆C}_{AA}+{∆C}_{FA}}{5{∆C}_{xyl}+C_{\left({NaHCO}_{3}\right)}}$$

(2) $$Carbon\;distribution\left(\%\right)=100\times \frac{n\times {∆C}_{pro}}{4{∆C}_{xyl}+C_{\left({NaHCO}_{3}\right)}}$$

where ∆C is the change in concentration; n is the number of carbon atoms in each compound (xylose = 5, SA = 4, FA = 1, AA = 2); pro denotes the product (SA, FA and AA); and inorganic carbon (HCO_3_^−^) is included. Carbon from gaseous CO_2_ supplementation was not included because its dissolution and consumption could not be quantitatively determined under the experimental conditions. Biomass was not explicitly accounted for, as cell growth was associated with biofilm formation and cannot be fully reflected in OD measurements.

## 3. Results and Discussions

As xylose is the most abundant sugar in hemicellulose, its microbial utilization is of particular importance. *A. succinogenes* can metabolize xylose, and therefore, the effect of electro-fermentation on SA production using xylose as the carbon source should be investigated.

Glucose and xylose enter central metabolism in *A. succinogenes* through distinct upstream routes but converge at the same downstream intermediates. Glucose is transported directly into the cell and metabolized via the Embden–Meyerhof–Parnas (glycolytic) pathway, generating phosphoenolpyruvate (PEP) and pyruvate that subsequently feed into either the C4 SA-producing pathway or the C3 branch, yielding FA, AA, and ethanol [23]. In contrast, xylose uptake involves conversion by xylose isomerase to xylulose, phosphorylation by xylulokinase to xylulose-5-phosphate, and entry into the pentose phosphate pathway. The resulting intermediates, such as glyceraldehyde-3-phosphate and fructose-6-phosphate, funnel into glycolysis and likewise converge at PEP and pyruvate [24]. Differences are expected due to the distinct uptake mechanisms of glucose and xylose, and these differences should be systematically characterized.

### 3.1. Effect of Applied Potential on SA, FA, and AA Yields at Varying Xylose Concentrations

**Figure 2:**
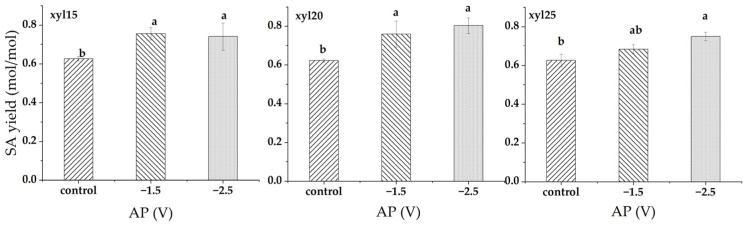
Succinic acid (SA) yield (mol/mol) at different applied potentials (APs). Conditions included a control without applied potential, −1.5 V, and −2.5 V. xyl15, xyl20, and xyl25 represent feed xylose levels of 15, 20, and 25 g/L, respectively. Bars represent the mean of triplicate experiments ± standard deviation. Different letters above the bars indicate significant differences at *p* < 0.05, while the same letter denotes no significant difference.

At xylose feed levels of 15, 20, and 25 g/L, applied potential significantly enhanced SA yield, though the extent varied with substrate concentration (Figure 2). At 15 g/L, SA yield increased from 0.627 mol/mol in the control to 0.756 mol/mol at −1.5 V (20.6% increase) and 0.741 mol/mol at −2.5 V (18.3% increase). Both potentials significantly improved the SA yield compared to the control, with no difference between them. At 20 g/L, SA yield rose from 0.623 mol/mol in the control to 0.759 mol/mol at −1.5 V (21.9% increase) and 0.803 mol/mol at −2.5 V (28.8% increase). At 25 g/L, SA yield increased from 0.626 mol/mol in the control to 0.684 mol/mol at −1.5 V (9.3% increase) and 0.749 mol/mol at −2.5 V (19.7% increase). The control and −2.5 V differed significantly, while −1.5 V showed no significant difference from either.

**Figure 3:**
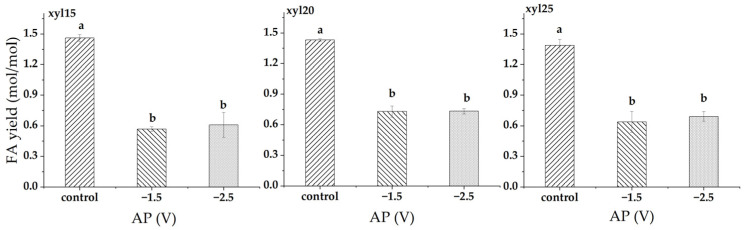
Formic acid (FA) yield (mol/mol) at different applied potentials (APs). Conditions included a control without applied potential, −1.5 V, and −2.5 V. xyl15, xyl20, and xyl25 represent feed xylose levels of 15, 20, and 25 g/L, respectively. Bars represent the mean of triplicate experiments ± standard deviation. Different letters above the bars indicate significant differences at *p* < 0.05, while the same letter denotes no significant difference.

At xylose feed levels of 15, 20, and 25 g/L, applied potential strongly suppressed FA yield (Figure 3). At 15 g/L, FA yield decreased from 1.461 in the control to 0.569 mol/mol at −1.5 V (61.1% decrease) and 0.609 mol/mol at −2.5 V (58.3% decrease). At 20 g/L, FA yield declined from 1.431 in the control to 0.729 mol/mol at −1.5 V (49.0% decrease) and 0.732 mol/mol at −2.5 V (48.8% decrease). At 25 g/L, FA yield fell from 1.388 in the control to 0.639 mol/mol at −1.5 V (54.0% decrease) and 0.692 mol/mol at −2.5 V (50.2% decrease). In all cases, the control was significantly higher than both applied potentials, while −1.5 V and −2.5 V did not differ significantly.

**Figure 4:**
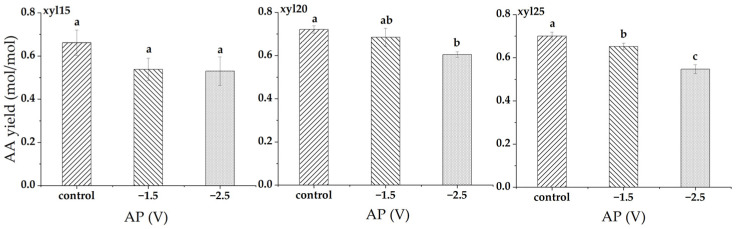
Acetic acid (AA) yield (mol/mol) at different applied potentials (AP). Conditions included a control without applied potential, −1.5 V, and −2.5 V. xyl15, xyl20, and xyl25 represent feed xylose levels of 15, 20, and 25 g/L, respectively. Bars represent the mean of triplicate experiments ± standard deviation. Different letters above the bars indicate significant differences at *p* < 0.05, while the same letter denotes no significant difference.

At xylose feed levels of 15, 20, and 25 g/L, applied potential had varying effects on AA yield (Figure 4). At 15 g/L, AA yield decreased from 0.662 in the control to 0.538 mol/mol at −1.5 V (18.7% decrease) and 0.530 mol/mol at −2.5 V (19.9% decrease), with no significant differences among them. At 20 g/L, AA yield declined from 0.721 in the control to 0.685 mol/mol at −1.5 V (5.0% decrease) and 0.605 mol/mol at −2.5 V (16.0% decrease). The control was significantly higher than −2.5 V, while −1.5 V did not differ from either. At 25 g/L, yield decreased from 0.700 in the control to 0.652 mol/mol at −1.5 V (6.9% decrease) and 0.547 mol/mol at −2.5 V (21.9% decrease), with all treatments significantly different from one another.

Applied potential consistently reduced FA and AA yield and redirected product distribution toward SA. At 15 and 20 g/L xylose, −1.5 V was sufficient to significantly enhance SA, whereas at 25 g/L, a stronger potential (−2.5 V) was required, which also provided additional suppression of AA.

**Figure 5:**
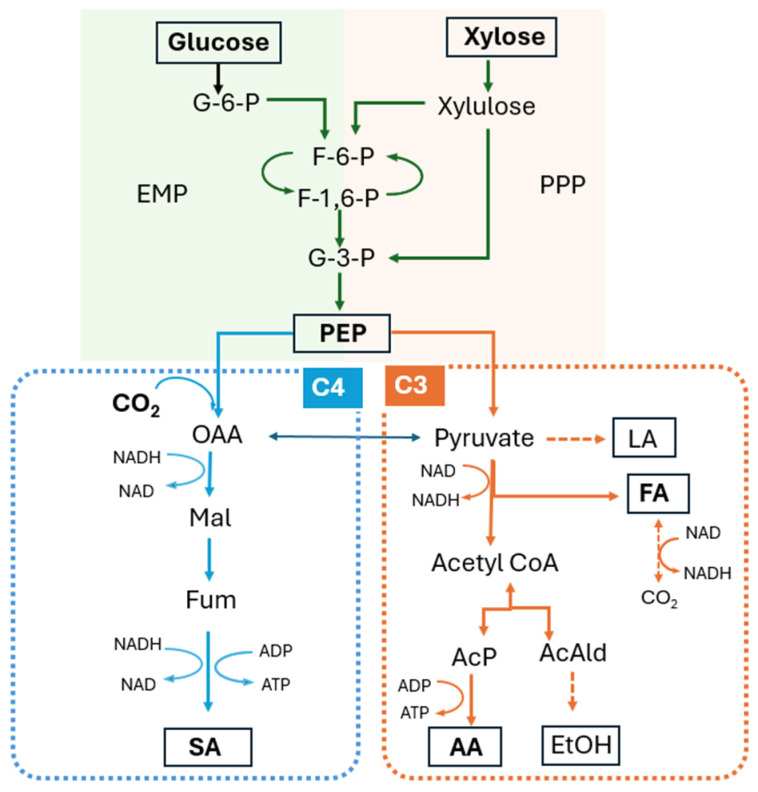
Simplified metabolic pathways for xylose metabolism and redox-dependent carbon flux distribution in *A. succinogenes*. Abbreviations: EMP, Embden–Meyerhof–Parnas pathway; PPP, pentose phosphate pathway; G-6-P, glucose-6-phosphate; F-6-P, fructose-6-phosphate; F-1,6-P, fructose-1,6-bisphosphate; G-3-P, glyceraldehyde-3-phosphate; PEP, phosphoenolpyruvate; OAA, oxaloacetate; Mal, malate; Fum, fumarate; SA, succinate; FA, formate; AA, acetate; LA, lactate; EtOH, ethanol; Acetyl CoA, acetyl coenzyme A; AcP, acetyl phosphate; AcAld, acetaldehyde.

This shift in product distribution can be interpreted from a redox perspective. Under electro-fermentation, the applied potential is expected to increase the intracellular reducing power by enhancing electron availability, thereby elevating the NADH/NAD^+^ ratio. Increased reducing equivalents favor the reductive C4 pathway, where phosphoenolpyruvate is carboxylated by phosphoenolpyruvate carboxykinase to oxaloacetate and subsequently reduced through malate and fumarate to succinic acid. In contrast, the formation of formate is associated with pyruvate formate-lyase activity within the C3 branch. When the intracellular redox state becomes more reduced, carbon flux is preferentially directed toward NADH-consuming reactions such as succinate formation, while flux through the C3 pathways decreases. A simplified metabolic scheme illustrating xylose metabolism, glycolysis, and the competing C4 and C3 branches is shown in Figure 5.

### 3.2. Effect of Applied Potential on SA, FA, and AA Titers at Varying Xylose Concentrations

**Figure 6:**
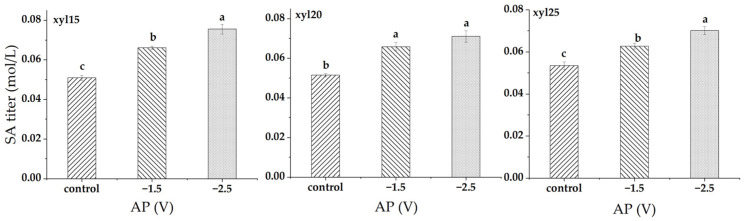
Succinic acid (SA) titers (mol/L) at different applied potentials (APs). Conditions included a control without applied potential, −1.5 V, and −2.5 V. xyl15, xyl20, and xyl25 represent feed xylose levels of 15, 20, and 25 g/L, respectively. Bars represent the mean of triplicate experiments ± standard deviation. Different letters above the bars indicate significant differences at *p* < 0.05, while the same letter denotes no significant difference.

**Figure 7:**
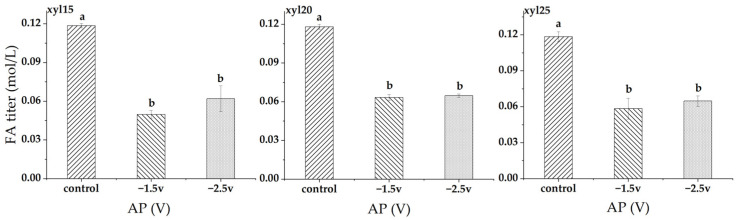
Formic acid (FA) titers (mol/L) at different applied potentials (AP). Conditions included a control without applied potential, −1.5 V, and −2.5 V. xyl15, xyl20, and xyl25 represent feed xylose levels of 15, 20, and 25 g/L, respectively. Bars represent the mean of triplicate experiments ± standard deviation. Different letters above the bars indicate significant differences at *p* < 0.05, while the same letter denotes no significant difference.

**Figure 8:**
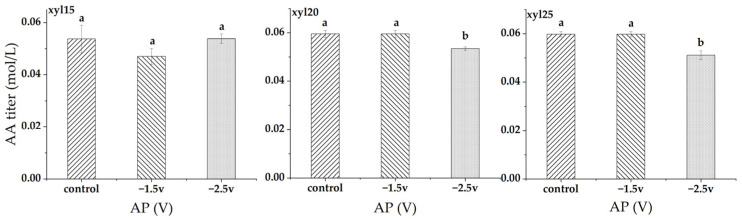
Acetic acid (AA) titers (mol/L) at different applied potentials (AP). Conditions included a control without applied potential, −1.5 V, and −2.5 V. xyl15, xyl20, and xyl25 represent feed xylose levels of 15, 20, and 25 g/L, respectively. Bars represent the mean of triplicate experiments ± standard deviation. Different letters above the bars indicate significant differences at *p* < 0.05, while the same letter denotes no significant difference.

At xylose feed levels of 15, 20, and 25 g/L, applied potential significantly enhanced SA titer (Figure 6). At 15 g/L, SA titer increased from 0.051 mol/L in the control to 0.066 mol/L at −1.5 V (29.9% increase) and 0.075 mol/L at −2.5 V (48.4% increase), with all treatments significantly different from one another. At 20 g/L, the SA titer rose from 0.051 mol/L in the control to 0.066 mol/L at −1.5 V (28.2% increase) and 0.071 mol/L at −2.5 V (38.3% increase); both potentials were significantly higher than the control, while no difference was observed between them. At 25 g/L, SA titer increased from 0.053 mol/L in the control to 0.063 mol/L at −1.5 V (17.3% increase) and 0.070 mol/L at −2.5 V (31.0% increase), with all treatments significantly different from one another. Comparing Figure 6 to Figure 2, it can be noted that the groups may not have the same letters (indicating the significance of differences is not the same between yield and titer). Succinate titer and yield describe different aspects of system performance and were analyzed as separate response variables. Succinate titer reflects the absolute amount of succinate accumulated in the culture (mol L^−1^), whereas succinate yield is normalized to xylose consumption (mol succinate per mol xylose). Differences in xylose uptake among voltage conditions alter the normalization used in yield calculations, which can change the relative separation among treatments even when titer trends are similar. In addition, yield incorporates variability from both succinate and xylose measurements, whereas titer depends primarily on succinate concentration. These differences in normalization and variance lead to different post hoc significance groupings between titer and yield. This explanation also applies to Figure 3 vs. Figure 7, and Figure 4 vs. Figure 8.

At xylose feed levels of 15, 20, and 25 g/L, applied potential consistently suppressed FA titer (Figure 7). At 15 g/L, FA titer decreased from 0.119 mol/L in the control to 0.050 mol/L at −1.5 V (57.9% decrease) and 0.062 mol/L at −2.5 V (47.8% decrease). At 20 g/L, FA titer declined from 0.118 mol/L in the control to 0.063 mol/L at −1.5 V (46.4% decrease) and 0.065 mol/L at −2.5 V (45.2% decrease). At 25 g/L, FA titer decreased from 0.118 mol/L in the control to 0.059 mol/L at −1.5 V (50.6% decrease) and 0.065 mol/L at −2.5 V (45.7% decrease). In all cases, the control was significantly higher than both applied potentials, while no difference was observed between −1.5 V and −2.5 V.

At xylose feed levels of 15, 20, and 25 g/L, applied potential had only minor effects on AA titer (Figure 8). At 15 g/L, AA titer remained nearly unchanged, ranging from 0.054 mol/L in the control to 0.047 mol/L at −1.5 V and 0.054 mol/L at −2.5 V, with no significant differences among treatments. At 20 g/L, the AA titer was stable at 0.059 mol/L in the control and −1.5 V, but decreased slightly to 0.054 mol/L at −2.5 V, which was significantly lower than the other two. At 25 g/L, the AA titer was also stable at 0.060 mol/L in the control and −1.5 V, but declined to 0.051 mol/L at −2.5 V, which was significantly lower than the control and −1.5 V.

In all, across all xylose feed levels, applied potential consistently increased SA titers, and suppressed FA, while AA remained largely stable with only minor decreases at −2.5 V. The greatest increase in SA occurred at 15 g/L, where titer rose from 0.051 mol/L in the control to 0.075 mol/L at −2.5 V. The strongest decrease in FA was also observed at 15 g/L, where titer declined from 0.119 mol/L in the control to 0.050 mol/L at −1.5 V. These results indicate that applied potential shifts carbon flux toward succinate production at the expense of FA, while AA titer is less affected.

### 3.3. Carbon Recovery and Balance Efficiency

To evaluate the completeness and efficiency of substrate conversion, carbon recovery was calculated by summing the carbon equivalents in measured products relative to the initial xylose consumed. This analysis integrated SA, FA, and AA as the dominant soluble products and provided an estimate of overall carbon closure under different applied potentials and substrate concentrations. Examining carbon recovery offers insight into whether applied potential primarily redirects flux among measured acids or also alters the extent of unaccounted carbon loss, such as to biomass, CO_2_, or undetected metabolites.

**Figure 9:**
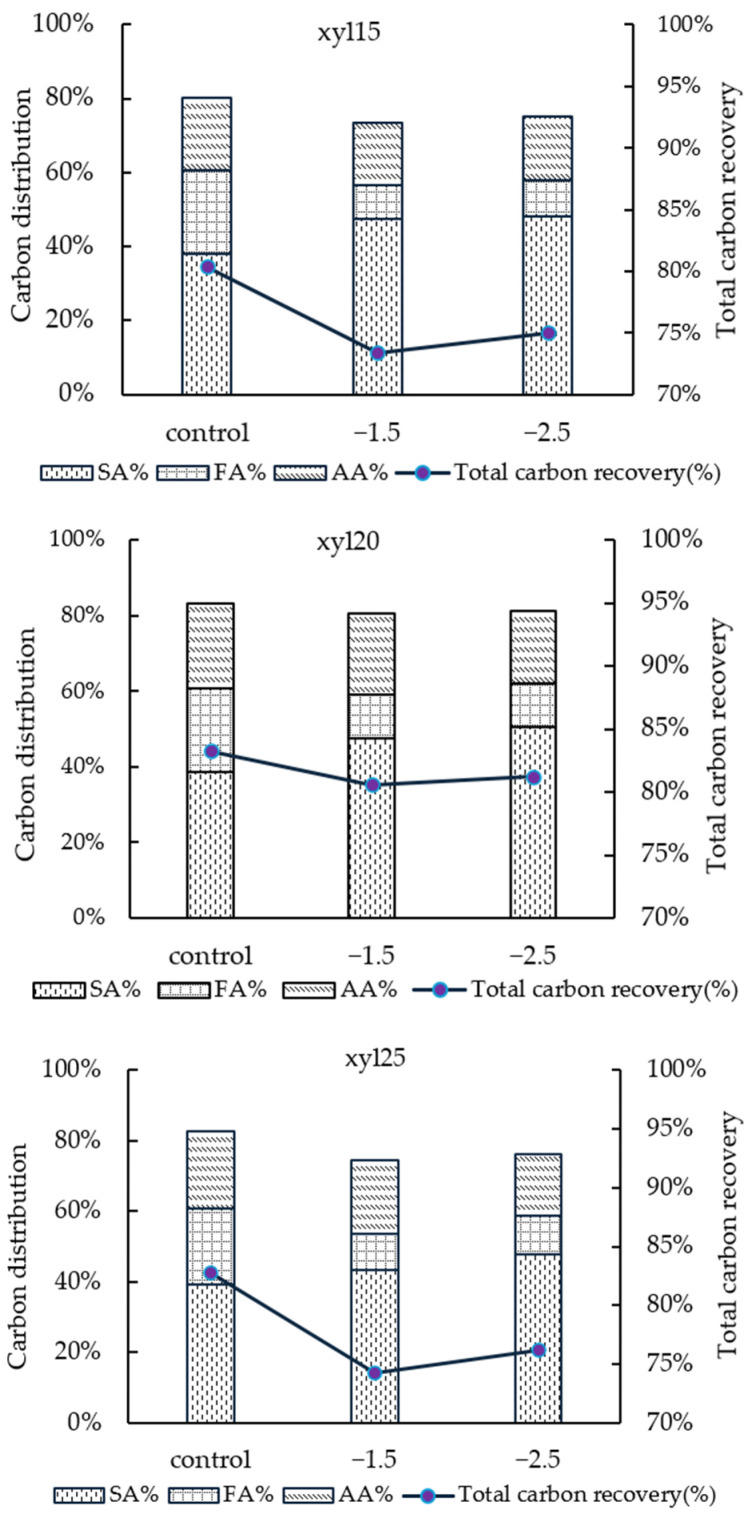
Carbon distribution and total carbon recovery at xylose (xyl) feed concentrations of 15, 20, and 25 g/L under different applied potentials, with calculations based on an initial NaHCO_3_ concentration of 10 g/L.

As shown in Figure 9, applied potential shifted product distribution toward SA at the expense of FA. This redistribution did not always translate into improved mass balance closure. With xylose, carbon recovery consistently declined despite increased SA allocation, decreasing from 80% to 75% at 15 g/L, from 83% to 81% at 20 g/L, and from 83% to 76% at 25 g/L. This indicates that additional carbon was not captured in FA or AA as measured products, suggesting diversion into alternative metabolic routes or incomplete recovery in the analyzed pools. The reduced carbon recovery is likely associated with several factors. First, xylose metabolism proceeds through the pentose phosphate pathway, which generates NADPH and may involve carbon recycling through the oxidative branch, resulting in additional CO_2_ release [24]. Second, applied potential alters intracellular redox balance, which may further increase carbon loss through CO_2_ evolution. Third, part of the substrate carbon may be incorporated into biomass and electrode-associated biofilm, which was not quantified in the carbon balance. Minor unmeasured metabolites may also contribute to the incomplete closure.

Such trends align with previous reports showing that SA yields from xylose are consistently lower than from glucose, with mass balance closures up to 18.2% lower under xylose fermentation [25]. Together, these results demonstrate that applied potential influences both product selectivity and overall carbon conservation, with outcomes dependent on substrate concentration.

### 3.4. Effect of CO_2_ Donors on SA Production Under EF

**Figure 10:**
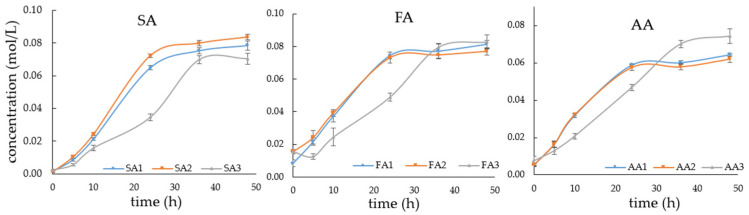
Time course of SA, FA, and AA concentrations (mol/L) with 15 g/L xylose at −2.5 V under three different conditions: 10 g/L NaHCO_3_ (SA1, FA1, AA1), 10 g/L NaHCO_3_ supplemented with gaseous CO_2_ (SA2, FA2, AA2), and 15 g/L NaHCO_3_ (SA3, FA3, AA3). Data illustrate metabolite accumulation patterns over 48 h; error bars represent standard deviation.

Beyond the influence of applied potential and carbon flux balance, the availability of inorganic carbon is another key determinant of SA formation, since the C4 pathway requires continuous bicarbonate (HCO_3_^−^) fixation via PEP carboxykinase [26,27]. To evaluate the effect of CO_2_ donors under electro-fermentation, additional experiments were performed at −2.5 V with 15 g/L xylose, comparing SA production with 10 g/L NaHCO_3_, 15 g/L NaHCO_3_, and a combination of 10 g/L NaHCO_3_ plus gaseous CO_2_ sparging. This setup allowed assessment of whether increasing bicarbonate concentration or supplementing with gaseous CO_2_ could further enhance SA yield relative to the baseline condition. Results are shown in Figure 10.

Relative to the baseline condition with 10 g/L NaHCO_3_, increasing bicarbonate supply to 15 g/L reduced the final SA concentration from 0.079 mol/L to 0.071 mol/L (statistically significant at the 95% confidence level, *p* < 0.05). At 15 g/L NaHCO_3_, SA accumulation was also delayed, reaching only 0.035 mol/L at 24 h compared with 0.065 mol/L and 0.072 mol/L under 10 g/L NaHCO_3_ and 10 g/L NaHCO_3_ with gaseous CO_2_, respectively. The SA plateau shifted to 36 to 48 h, reflecting slower metabolic activity under elevated ionic strength. This is likely due to osmotic stress and ionic burden, which hinder early carboxylation flux and delay SA accumulation. The inhibitory effect of excessive NaHCO_3_ is consistent with previous reports showing that high osmolarity suppresses both growth and SA synthesis in *A. succinogenes*, though this inhibition can be alleviated by osmoprotectants such as proline [28]. These results show that bicarbonate supply does not linearly enhance SA production. Concentrations beyond the optimal range impair metabolic efficiency. Previous studies reported that carbonate concentrations ranging from 0 to 500 mM enhanced succinic acid production, with yields increasing as the concentration increased up to an optimum of approximately 400 mM. Further increases beyond this level did not improve SA production, indicating that excessive bicarbonate does not provide additional benefits once CO_2_ availability becomes sufficient [29].

As can be seen from Table 1, CO_2_ sparging with 10 g/L NaHCO_3_ enhanced SA production, yielding 0.852 mol/mol with the highest carbon recovery (83.79%, excluding gaseous CO_2_ input). Carbon recovery was calculated based on the carbon from consumed xylose and the inorganic carbon initially supplied as NaHCO_3_; residual dissolved inorganic carbon was not directly measured, which may introduce some uncertainty in the calculated values. Carbon distribution analysis showed that CO_2_ supplementation increased flux toward SA (54.70%) compared with 47.79% under 10 g/L NaHCO_3_ alone and 37.33% under 15 g/L NaHCO_3_. In contrast, FA remained low (10.20%) and AA moderate (18.89%). These results indicate that CO_2_ functions as a co-substrate with NaHCO_3_, consistent with a stoichiometric requirement of 1 mol CO_2_ per mol SA. Because excessive NaHCO_3_ imposes osmotic inhibition, direct CO_2_ sparging offers an effective strategy to expand substrate availability for carboxylation without the drawbacks of high bicarbonate concentrations.

Overall, NaHCO_3_ played a dual role during fermentation. First, it served as a co-substrate, supplying inorganic carbon for carboxylation reactions that drive SA formation. In *A. succinogenes*, bicarbonate reacts with phosphoenolpyruvate (PEP) to form oxaloacetate, which is sequentially reduced through malate and fumarate to SA [23]. The availability of CO_2_/HCO_3_^−^ thereby facilitates carbon flux distribution between competing pathways: high CO_2_ availability promotes flux through phosphoenolpyruvate carboxykinase (PEPCK) and the C4 branch, favoring SA formation, while limiting diversion toward C3 by-products such as FA, AA, and ethanol [30]. Second, NaHCO_3_ acted as a buffer. At near-neutral pH, bicarbonate ions neutralize protons released during organic acid formation, thereby stabilizing extracellular pH and supporting sustained bacterial activity and balanced redox metabolism [29].

## 4. Conclusions

Applied potential was effective in enhancing SA selectivity in xylose-fed systems, but the extent of improvement depended on feed concentration. The xylose-fed system showed consistent recovery losses under both −1.5 V and −2.5 V despite improved SA yields. Supplementation with gaseous CO_2_ alongside bicarbonate further enhanced SA accumulation and carbon recovery when xylose was used as the substrate, underscoring the importance of inorganic carbon availability. Overall, these findings demonstrated that electro-fermentation performance was influenced by both substrate concentration and inorganic carbon supply. Optimizing SA production will require integrating external reducing power with carbonate strategies tailored to the primary carbon source.

Future work should focus on transcriptomic or metabolic analyses to elucidate the cellular response to applied potential and CO_2_ availability, as well as fed-batch electro-fermentation and scale-up studies to evaluate process stability and industrial feasibility. Further studies involving direct measurement of CO_2_ evolution and biomass formation are needed to better reveal the carbon balance.

## Figures and Tables

**Table 1:** Product yield (mol/mol), carbon distribution, and total carbon recovery from xylose 15 g/L at −2.5 V under different CO_2_ supply conditions. Conditions tested were 10 g/L NaHCO_3_, 15 g/L NaHCO_3_, and 10 g/L NaHCO_3_ with gaseous CO_2_ supplementation. Values are presented as mean ± standard deviation (n = 3). Different superscript letters within the same column indicate statistically significant differences among conditions based on one-way ANOVA (*p* < 0.05).

		Yield (mol/mol)			Carbon Distribution			Total Carbon Recovery
	Product	SA	FA	AA	SA	FA	AA	
CO_2_ Supply								
NaHCO_3_ 10 g/L		0.736 ± 0.012 ^b^	0.604 ± 0.007 ^b^	0.525 ± 0.015 ^b^	47.79% ± 1.12 ^b^	9.80% ± 0.23 ^ab^	17.04% ± 0.07 ^b^	74.63% ± 1.31 ^b^
NaHCO_3_ 15 g/L		0.616 ± 0.018 ^c^	0.618 ± 0.015 ^b^	0.593 ± 0.035 ^a^	37.33% ± 1.96 ^c^	9.37% ± 0.66 ^b^	17.97% ± 1.20 ^a^	64.67% ± 3.86 ^c^
NaHCO_3_ 10 g/L + CO_2_		0.852 ± 0.007 ^a^	0.636 ± 0.006 ^a^	0.589 ± 0.013 ^a^	54.70% ± 0.42 ^a^	10.20% ± 0.79 ^a^	18.89% ± 0.12 ^a^	83.79% ± 0.36 ^a^

## Data Availability

The original contributions presented in this study are included in the article. Further inquiries can be directed to the corresponding author.
